# Malignant mesothelioma in construction workers: the Apulia regional mesothelioma register, Southern Italy

**DOI:** 10.1186/s13104-019-4675-4

**Published:** 2019-09-30

**Authors:** L. Vimercati, D. Cavone, A. Caputi, M. C. Delfino, L. De Maria, G. M. Ferri, G. Serio

**Affiliations:** 1 School of Medicine, Interdisciplinary Department of Medicine (DIM), Ramazzini Occupational Medicine Section, “Policlinico” University Hospital, UOC of Occupational Medicine, University of Bari “A. Moro”, Piazza G, Cesare 11, 70124 Bari, Italy; 2 School of Medicine, Department of Emergency and Organ Transplantation (DETO), Division of Pathology, University of Bari “A. Moro”, Piazza G, Cesare 11, 70124 Bari, Italy

**Keywords:** Asbestos, Construction, Workers, Malignant mesothelioma, Compensation, Work-related cancer, Mesothelioma register

## Abstract

**Objective:**

Asbestos was widely used in construction in both a friable and a compact form until the 1990s, before its use was banned. Today, many of these materials are still in situ and represent a source of risk for construction workers. The objective of the study was to analyse the cases of mesothelioma arising among construction workers registered in the Apulia regional register of mesothelioma.

**Results:**

For the period 1993–2018, there were 178 male cases, and 10.2% of the cases were present in the regional register. The average age at diagnosis was 64.7 years. The site was pleural in 96.06% of cases, with a diagnosis of certainty in 86.5% of the total cases and 61.8% of cases with epithelial histology. The average latency is 43.9 years. In 75.2% of cases, the exposure began between 1941 and 1970, with an average duration of 24.3 years. The age at the start of exposure in 68.5% of cases is between 8 and 20 years. The ORs were 2.5 (C.I. 95% 1.01–6.17) for the epithelioid histotype and the high duration of exposure. The data underline the need for prevention and information on all activities involving construction workers in which asbestos-containing materials are still used.

## Introduction

Construction is one of the most dangerous industries worldwide. The health risks of construction workers depend on job tasks, each with a specific associated risk and exposure, but these workers are also passively exposed to risks produced by nearby co-workers [1–3]. There are many known carcinogens used in the construction industry [1]. Moreover, work injury and specific exposures to disease are important risk factors for the development of chronic diseases among construction workers given the nature of the work (temporary, manual and outdoors), as well as among other workers not in the construction sector who are exposed to the same risks [4–18].

Asbestos has been widely used in construction, and a series of reports have been produced on asbestos-related diseases in these workers. These workers may often be unaware of their exposure to asbestos, as in the case of indirect exposure among certain types of workers, such as carpenters, electricians, plumbers, and welders, if they were on-site during insulation activities [19].

An increased risk of mesothelioma and asbestos-related pleural disease was reported in construction workers [20–25], and the adjusted odds ratios for lung cancer and mesothelioma were also described among workers who engaged in high levels of smoking [26–29]. Engholm [30] reported an SIR of 3.16 statistically significant (SS) for pleural mesothelioma among Swedish construction workers. In a cohort of construction workers, Dement [31] found an SMR of 5.93 SS for mesothelioma. An updated follow–up of this cohort [32] found an SMR of 6.86 SS for mesothelioma. Rolland [33] found a high risk of pleural mesothelioma among construction workers other than insulators, and especially for plumbers and pipe fitters, bricklayers, tile setters, carpenters and other construction workers not elsewhere classified (n.e.c.), with an OR of 3.46 SS. Stocks [34] found mesothelioma SRRs of 7.1 SS for male UK construction industry workers aged below 65 years. The same author [35] found a significantly increased incidence in pipe fitters (4.5), electrical workers (2.7), plumbing and heating engineers (2.3), carpenters and joiners (2.7), scaffolders (12) and labourers (n.e.c. (3.3). Järvholm [36] reported that in a cohort of 367,568 Swedish construction workers, there were 419 cases of pleural mesothelioma between 1972 and 2009. In a cohort of 17,345 sheet metal workers, Welch [37] found an overall mortality that included excess mortality for mesothelioma, with an SMR of 7.34 SS. In Belgium, the mesothelioma mortality was reported to be significantly high among manual workers in the construction sector (SMR 227) [38]. In a study on compensation for ARD in Japan, Sawanyawisuth [39] found that construction had the highest compensated risk ratio.

An [40] reported high ARD claims made to the Korea Workers’ Compensation and Welfare Service in the construction sector. Tomasallo [41] found increased odds of mesothelioma in Wisconsin among construction workers.

Peritoneal mesothelioma in the construction industry in Sweden was described by Malkerr in [42], especially among insulation workers, and by Fonte [43] in a case involving workers in the building industry aged 14–55 years who had been cutting and shaping asbestos-containing cement panels. Long-term exposures can induce peritoneal mesothelioma, even in subjects with environmental exposures [44]. Because there is general consensus that malignant mesothelioma (MM) can develop even after short exposures to very low levels of asbestos, there is no evidence of a threshold level below which there is no risk and that the risk increases with the intensity and duration of exposure [45–47].

In this study, we analyse the cases of mesothelioma arising among construction workers registered in the Apulia regional register of mesothelioma.

The regional register records the mesothelioma cases starting in 1993 according to the national guidelines of the RENAM (Italian National Register of Malignant Mesotheliomas). All cases underwent diagnostic revision. The reconstruction of the exposure was performed by means of direct interviews with the subjects or, in the event of death, with the family members using the standardized RENAM questionnaire [48, 49]. The exposures were classified according to the Istat ATECO 91 classification [50]. Only those with code 45 were analysed, including all sub-sectors, sectors and tasks. For subjects exposed in more than one work sector, the prevailing sector was considered to provide a longer duration and level of exposure. For all subjects, all other possible exposures to asbestos, including environmental, residential, familial, leisure and non-working exposures, were investigated and excluded. Stata 12 software was used for statistical analysis, univariate analysis and the study of the associations between types of mesothelioma and different variables. Score tests for trend and homogeneity tests were also carried out.

## Main text

The characteristics of mesothelioma cases in the Apulia regional register are reported in Table 1.

**Table 1:** Characteristics of the male mesothelioma cases in the Apulia regional register

Year of diagnosis	Sex (male)	%
1980–1989	8	4.5
1990–1995	11	6.2
1996–2000	28	15.7
2001–2005	44	24.7
2006–2010	48	26.9
2011–2015	25	11.04
2016–2018	14	7.8
Total	178	100
Anatomical site		
Pleura	171	96.06
Peritoneum	6	3.3
Pericardium	1	0.5
Total	178	100
Histological type		
Epithelioid	110	61.8
Fibrous	14	7.8
Biphasic	19	10.7
NOS	45	25.2
Total	178	100
Age at diagnosis (years)		
35–40	4	2.2
41–50	8	4.5
51–60	51	28.6
61–70	63	35.4
71–80	46	25.8
81–ω	6	3.3
Total	178	100
Year start exposure		
1931–1940	13	7.3
1941–1950	29	16.2
1951–1960	49	27.5
1961–1970	56	31.5
1971–1980	14	7.8
1981–1990	9	5.05
1991–2000	8	4.5
Total	178	100
Exposure duration (years)		
0–1	2	1.1
1–10	56	31.5
11–20	23	12.9
21–30	26	14.6
31–40	34	19.1
41–50	30	16.8
51–60	7	3.9
Total	178	100
Age at beginning of exposure		
8–14	43	24.1
15–20	79	44.4
21–30	38	21.3
31–40	10	5.6
41–50	6	3.3
51–ω	2	1.1
Total	178	100
Latency (years)		
11–20	11	6.2
21–30	15	8.4
31–40	45	25.2
41–50	39	21.9
51–60	49	27.5
61–70	17	9.5
71–ω	2	1.1
Total	178	100

The number of cases in the construction sector with exposure periods until 2018 was 178, all occurring in male subjects (10.2% of the cases present in the regional register).

The age at diagnosis, the average of which was 64.7 years, was 51–80 years in 89.8% of cases. The site was pleural in 96.06%, with a diagnosis of certainty in 86.5% of the total cases and 61.8% with epithelial histology.

The average latency is 43.9 years, with 83.1% of cases having an latency between 21 and 60 years. In 75.2% the exposure began between 1941 and 1970 and had an average duration of 24.3 years (between 1 and 10 years in 31.5% of cases). The date of initial exposure correlates with the peak of incident cases (years 2001–2010), equal to 51.6% of cases registered in the sector.

The age at the start of exposure in 68.5% is between 8 and 20 years. The exposure was confirmed via direct interview with the subject in 44.9%; in 61.2%, the exposure was classified as occupationally certain or probable, and in only 38.7% of cases, the exposure was classified as possible.

In total, 21.9% of the respondents reported having always worked in the construction sector, both in Italy and abroad in Switzerland, France, Germany, Belgium, Libya, Ghana, Venezuela and the USA. Regarding the analysis of the tasks performed and declared during the interview, 48 subjects reported having worked with products containing asbestos (26.9%). Eighty respondents reported that they carried out the generic job of mason or handyman bricklayer (44.9%). The remaining 50 respondents played various roles, such as tiler, plasterer, building carpenter, plumber, and electrician.

From the analysis of the risk distribution (ORs) of the histotype according to the duration of exposure, latency and age at diagnosis, an OR of 2.5 (C.I. 95% 1.01–6.17) was found for the epithelioid histotype and the high duration of exposure (Table 2).

**Table 2:** Risk (ORs) distribution of the different histological types of mesothelioma in construction workers by exposure length, latency period and age at diagnosis

Histological type	Variables	Levels (years)	Case	Controls	Odds ratios (LCL–UCL*)	p**
Epithelioid	Exposure length	≤ 6	20	20	1.00 (0.00–0.00)	
		7–32	47	30	1.56 (0.73–3.37)	
		> 32	30	12	2.50 (1.01–6.17)	0.04
	Latency period	≤ 34	24	13	1.00 (0.00–0.00)	
		35–53	45	35	0.69 (0.31–1.54)	
		> 53	28	14	1.08 (0.43–2.42)	0.82
	Age at diagnosis	< 57	24	15	1.00 (0.00–0.00)	
		57–70	46	29	0.99 (0.45–2.18)	
		> 70	27	18	0.93 (0.39–2.23)	0.88
NOS	Exposure length	≤ 6	4	36	1.00 (0.00–0.00)	
		7–32	4	73	0.49 (0.12–1.91)	
		> 32	1	41	0.21 (0.00–1.55)	0.13
	Latency period	≤ 34	3	34	1.00 (0.00–0.00)	
		35–53	5	75	0.75 (0.18–3.02)	
		> 53	1	41	0.27 (0.00–2.05)	0.26
	Age at diagnosis	< 57	3	36	1.00 (0.00–0.00)	
		57–70	4	71	0.68 (0.15–2.03)	
		> 70	2	43	0.55 (0.10–1.80)	0.52
Biphasic	Exposure length	≤ 6	5	35	1.00 (0.00–0.00)	
		7–32	9	68	0.92 (0.30–2.83)	
		> 32	4	38	0.73 (0.19–2.76)	0.67
	Latency period	≤ 34	4	33	1.00 (0.00–0.00)	
		35–53	9	71	1.04 (0.31–3.43)	
		> 53	5	37	1.11 (0.29–4.17)	0.87
	Age at diagnosis	< 57	4	35	1.00 (0.00–0.00)	
		57–70	9	66	1.19 (0.36–3.91)	
		> 70	5	40	1.09 (0.29–4.07)	0.91
Fibrous	Exposure length	≤ 6	3	37	1.00 (0.00–0.00)	
		7–32	5	72	0.85 (0.21–3.42)	
		> 32	2	40	0.61 (0.11–3.28)	0.60
	Latency period	≤ 34	2	35	1.00 (0.00–0.00)	
		35–53	6	74	1.41 (0.30–2.20)	
		> 53	2	40	0.87 (0.14–1.80)	0.88
	Age at diagnosis	< 57	1	38	1.00 (0.00–0.00)	
		57–70	5	70	2.71 (0.39–4.45)	
		> 70	4	41	3.70 (0.52–5.45)	0.23

*Lower and Upper 95% Cornefield confidence levels

**Probability of Test Chi square^2^ for trend

An increased duration of exposure and risk of developing the disease occurred with the more frequent epithelioid histotype, confirming a significant positive trend. Positive trend results were also found for fibrous histotype and age at diagnosis, although this finding was not SS.

In the RENAM for the years 1993/2015, the building with 15.5% of the total ascertained exposures is the first economic category for exposure frequency, with 3002 cases [51, 52].

In Italy, Carex [53] estimated approximately 70,000 exposures in the construction sector at the beginning of the 2000s. Additionally, the 2017 data of the Italian Institute of Statistics (ISTAT) report 1,309,649 employees in the construction sector [54], and estimates of materials containing asbestos (MCA) in situ were approximately 32 million tons for disposal [55]. It should be emphasized that all these workers may have undergone passive exposure to asbestos during processing. In addition, in the construction sites, the general task of mason/unskilled worker provides for a great interchangeability of tasks/tasks in practice. In this regard, we must emphasize that 24.1% of the cases reported here began working between 8 and 15 years without any training/information on risks. This figure can also explain 6.7% of our cases diagnosed between 35 and 50 years of age.

In Italy, other regional mesothelioma registries have reported an excessive number of MM in the construction sector. Merler [56] in Veneto reported a high prevalence of mesothelioma with a rate of 4.1 × 10^5^ × year SS. Regarding the latency period, none of the cases reported here have a latency of less than 11 years from first exposure, which is consistent with previous literature. The Helsinki Criteria Consensus report in 1997 established that a minimum of 10 years from the first exposure is required to attribute MM to asbestos exposure [57]. None of the eight cases, with the start of exposure in the years 1991–2000, reported having been aware of the possible exposure to asbestos. In this group the average latency was 15.5 years (range 11–19), all were employed in maintenance operations and building renovations in civilian homes.

Romeo [58] reported the results of the Lazio register with the highest number of cases among construction workers. D’Agostin [59] reported data from the Friuli Venezia Giulia register, where cases in the construction sector were 12.2% of all cases registered and had a latency of 46.3 years. Our latency result, 43.9 years, is in accordance with Friuli Venezia Giulia register report, as well as the percentage of cases occupied in construction compared to the total number of cases in our register, 10.2%.

Regarding the cases presented here, 6 peritoneal mesotheliomas, with a media duration of exposure of 19 years, and a pericardial mesothelioma, a subject who had worked in the sector for 16 years in the 1950s and 1960s, none of these seven cases had particular exposure conditions. Except for two peritoneal cases that had always worked in construction, even abroad, in Switzerland for 8 years, in Libya for 3 years and in France for 1 year in the 1960s. Two other peritoneal cases had worked for over 25 years since the 1980s and another for 45 years since the 1960s. To emphasize that none of these seven patients had awareness and remember to have been in contact with asbestos or MCA.

In general, our finding that approximately 10% of all regional cases are due to asbestos exposure in the construction sector is in agreement not only with Italian data [51, 52] but also with South Korean [60] and German findings [29, 61].

## Limitations

A limitation of this study is the lack of analysis of single sectors and risk profiles, which may have differed in intensity, duration, materials used and type of fibre, given the low number of subjects present in our database for each specific task. Conversely, in the literature, the risks were primarily detected in specialized trades, such as insulation workers and plumbers [1], and the main high-risk group among construction workers are considered insulators, carpenters, plumbers and electricians [1, 19, 33, 62–64]. Moreover, Jarvholm [1] noted that the occurrence of pleural mesothelioma among Swedish construction workers showed that secondary exposure or indirect exposures could be the cause of a majority of the mesothelioma cases, which is in accordance with our results on the tasks performed in the cases reported here.

Current occupational exposure among construction workers occurs during the maintenance and remediation of asbestos-containing buildings [19, 65]. Asbestos in older buildings may also be an environmental exposure for occupants and residents during maintenance, renovation and demolition in homes, schools, workplaces and public buildings [66, 67].

In Italy, despite the asbestos ban since 1992 (law 257/92), occupational asbestos exposure is still a risk for construction workers, especially if controls are insufficient to protect construction workers or if asbestos removal or waste disposal workers do not take adequate precautions [19, 68, 69]. Investigations of occupational risk factors among construction workers and evaluations of preventive practices are needed to prevent the occurrence of mesothelioma in this hazardous occupation. For example, the early diagnosis of mesothelioma is considered to be important for prognosis, and mesothelin may be useful as a blood tumour marker in the early diagnosis of mesothelioma in a mass examination [70, 71], as well as to identify genetic imbalances that could play an important role in the initial development of mesothelioma [72].

These data underline the need for prevention and information on all activities involving construction workers where MCA are still in situ but may be unknown or ignored among these workers.

## Data Availability

Data will be provided upon request to Luigi Vimercati (MD, PhD), Interdisciplinary Department of Medicine, Occupational Medicine “B. Ramazzini”, University of Bari Medical School, 70124 Bari, Italy. E-mail: luigi.vimercati@uniba.it

## Abbreviations

MM: malignant mesothelioma
SS: statistically significant
n.e.c.: not elsewhere classified
ARD: asbestos-related diseases
RENAM: Italian National Register of Malignant Mesotheliomas
ISTAT: Italian Institute of Statistics
MCA: materials containing asbestos
